# 2-(2,4,5-Trimeth­oxy­phen­yl)-2,3-dihydro­quinolin-4(1*H*)-one

**DOI:** 10.1107/S1600536812002917

**Published:** 2012-02-10

**Authors:** Suchada Chantrapromma, Pumsak Ruanwas, Nawong Boonnak, Kan Chantrapromma, Hoong-Kun Fun

**Affiliations:** a Crystal Materials Research Unit, Department of Chemistry, Faculty of Science, Prince of Songkla University, Hat-Yai, Songkhla 90112, Thailand; b Research Unit of Natural Products Utilization, Walailak University, Thasala, Nakhon Si Thammarat 80160, Thailand; c X-ray Crystallography Unit, School of Physics, Universiti Sains Malaysia, 11800 USM, Penang, Malaysia

## Abstract

In the title aza-flavanone, C_18_H_19_NO_4_, an intra­molecular cyclization product of chalcone, the central heterocyclic ring is in an envelope conformation and the dihedral angle between the benzene rings is 51.03 (10)°. The meth­oxy groups at the *ortho* and *para* positions are slightly twisted from the benzene ring to which they are bound [C—O—C—C = 21.9 (3) and −171.93 (18)°, respectively], whereas the meth­oxy group at the *meta* position is almost coplanar [C—O—C—C = 3.5 (3)°]. In the crystal, mol­ecules are linked by N—H⋯O hydrogen bonds and weak C—H⋯O inter­actions into chains along the [001] direction. Weak C—H⋯π inter­actions also occur.

## Related literature

For background to the syntheses and properties of aza-flavanones, see: Göker *et al.* (2005 ▶); Xia *et al.* (1998 ▶). For ring conformations, see Cremer & Pople (1975 ▶). For the stability of the temperature controller used in the data collection, see Cosier & Glazer, (1986 ▶).
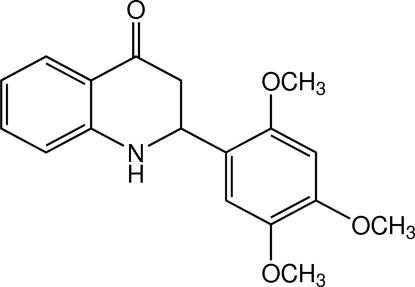


## Experimental

### 

#### Crystal data

C_18_H_19_NO_4_
*M* *_r_* = 313.34
Monoclinic, 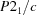

*a* = 10.7354 (11) Å
*b* = 17.1525 (16) Å
*c* = 8.6471 (8) Å
β = 102.981 (2)°
*V* = 1551.6 (3) Å^3^
*Z* = 4
Mo *K*α radiation
μ = 0.10 mm^−1^
*T* = 100 K
0.41 × 0.16 × 0.06 mm

#### Data collection

Bruker APEXII CCD diffractometer
Absorption correction: multi-scan (*SADABS*; Bruker, 2005 ▶) *T*_min_ = 0.962, *T*_max_ = 0.994
13335 measured reflections
4511 independent reflections
2751 reflections with *I* > 2σ(*I*)
*R*_int_ = 0.062

#### Refinement

*R*[*F* ^2^ > 2σ(*F* ^2^)] = 0.063
*wR*(*F* ^2^) = 0.156
*S* = 1.03
4511 reflections
215 parameters
H atoms treated by a mixture of independent and constrained refinement
Δρ_max_ = 0.38 e Å^−3^
Δρ_min_ = −0.30 e Å^−3^

### 

Data collection: *APEX2* (Bruker, 2005 ▶); cell refinement: *SAINT* (Bruker, 2005 ▶); data reduction: *SAINT*; program(s) used to solve structure: *SHELXTL* (Sheldrick, 2008 ▶); program(s) used to refine structure: *SHELXTL*; molecular graphics: *SHELXTL*; software used to prepare material for publication: *SHELXTL* and *PLATON* (Spek, 2009 ▶).

## Supplementary Material

Crystal structure: contains datablock(s) global, I. DOI: 10.1107/S1600536812002917/hb6602sup1.cif

Structure factors: contains datablock(s) I. DOI: 10.1107/S1600536812002917/hb6602Isup2.hkl

Supplementary material file. DOI: 10.1107/S1600536812002917/hb6602Isup3.cml

Additional supplementary materials:  crystallographic information; 3D view; checkCIF report

## Figures and Tables

**Table 1:** Hydrogen-bond geometry (Å, °) *Cg*1 and *Cg*2 are the centroids of the C1–C6 and C10–C15 rings, respectively.

*D*—H⋯*A*	*D*—H	H⋯*A*	*D*⋯*A*	*D*—H⋯*A*
N1—H1N1⋯O3^i^	0.90 (3)	2.32 (3)	3.156 (2)	155 (3)
C2—H2*A*⋯O4^i^	0.95	2.59	3.439 (3)	150
C16—H16*B*⋯O3^ii^	0.98	2.58	3.459 (3)	150
C8—H8*B*⋯*Cg*1^iii^	0.99	2.74	3.698 (2)	164
C16—H16*C*⋯*Cg*1^iv^	0.98	2.68	3.518 (3)	144
C17—H17*C*⋯*Cg*2^ii^	0.98	2.76	3.560 (3)	140
C18—H18*C*⋯*Cg*2^i^	0.98	2.75	3.574 (3)	142

Symmetry codes: (i) 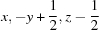; (ii) 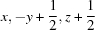; (iii) 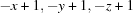; (iv) 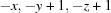.

**Fig. 1.**
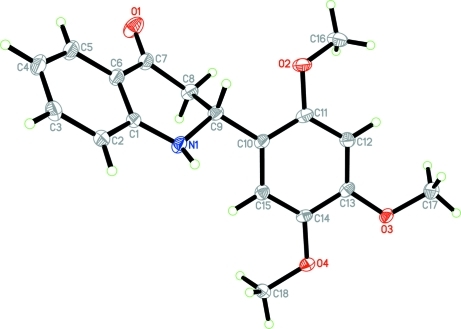
The molecular structure of the title compound, showing 50% probability displacement ellipsoids.

**Fig. 2.**
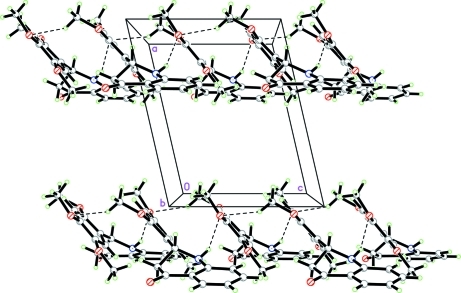
Partial packing diagram of the title compound viewed approximately along the b axis, showing chains running along the c axis. Hydrogen bonds are shown as dashed lines.

## supplementary crystallographic information

### Comment

Aza-flavanone or 2-aryl-2,3-dihydroquinolin-4(1*H*)-one, a synthesized analogue of flavanone, can be achieved by intramolecular cyclization of a chalcone derivative in basic medium (Xia *et al.*, 1998). They are also found to exhibit antibacterial, antifungal (Göker *et al.*, 2005) and anticancer activities (Xia *et al.*, 1998). In the course of our research on medicinal chemistry, we have synthesized the title aza-flavanone (I) in order to study its biological activity.

The total molecule of (I) is twisted (Fig. 1). The dihedral angle between two benzene rings is 51.03 (10)°. The N-atom containing central ring is in an envelope conformation with the puckered C9 atom having the maximum deviation of 0.352 (2) Å, and the puckering parameter Q = 0.502 (2) Å, θ = 124.5 (2)° and φ = 110.8 (3)° (Cremer & Pople, 1975). The three methoxy groups of the 2,4,5-trimethoxyphenyl unit have two different orientations: the two methoxy groups at *ortho* (at atom C11) and *para* (at atom C13) positions are slightly twisted from the attached benzene ring with torsion angles C16—O2—C11—C12 = 21.9 (3)° and C17—O3—C13—C14 = -171.93 (18)°, whereas the third one at *meta* (at atom C14) position is co-planar with the torsion angle of C18—O4—C14—C15 = 3.5 (3)°. These angle values also indicated that the methyl group at *para* position points towards the *ortho*-methoxy but points away from the *meta*-methoxy due to the steric effect.

In the crystal (Fig. 2), the molecules are linked by N—H···O hydrogen bonds and weak C—H···O interactions (Table 1) into chains along the *c* axis. C—H···π interactions (Table 1) also occur.

### Experimental

To a 50 ml round-bottom flask filled with 2,4,5-trimethoxybenzaldehyde (0.50 g, 2.55 mmol), EtOH (20 ml) and 2-aminoacetophenone (0.31 ml, 2.55 mmol) were sequentially added at room temperature. After stirring for a while, 5 ml of 30% NaOH (aq) was added slowly and was then further stirred for 2 h. A pale yellow precipitate was formed and collected by filtration yielding the title compound (I) (1.26 g, 75% yield), which was further recrystallized in EtOH to obtain yellow needles of (I) after several days, m.p. 419–420 K.

### Refinement

Amide H atom was located in a Fourier difference map and refined isotropically. The remaining H atoms were positioned geometrically and allowed to ride on their parent atoms, with C—H = 0.95 Å for aromatic, 1.00 for CH, 0.99 Å for CH_2_ and 0.98 Å for CH_3_ atoms. The *U*_iso_ values were constrained to be 1.5*U*_eq_ of the carrier atom for methyl H atoms and 1.2*U*_eq_ for the remaining H atoms. A rotating group model was used for the methyl groups.

### Crystal data

**Table:**

C_18_H_19_NO_4_	*F*(000) = 664
*M**_r_* = 313.34	*D*_x_ = 1.341 Mg m^−^^3^
Monoclinic, *P*2_1_/*c*	Melting point = 419–420 K
Hall symbol: -P 2ybc	Mo *K*α radiation, λ = 0.71073 Å
*a* = 10.7354 (11) Å	Cell parameters from 4511 reflections
*b* = 17.1525 (16) Å	θ = 2.0–30.0°
*c* = 8.6471 (8) Å	µ = 0.10 mm^−^^1^
β = 102.981 (2)°	*T* = 100 K
*V* = 1551.6 (3) Å^3^	Needle, yellow
*Z* = 4	0.41 × 0.16 × 0.06 mm

### Data collection

**Table:**

Bruker APEXII CCD diffractometer	4511 independent reflections
Radiation source: sealed tube	2751 reflections with *I* > 2σ(*I*)
Graphite monochromator	*R*_int_ = 0.062
φ and ω scans	θ_max_ = 30.0°, θ_min_ = 2.0°
Absorption correction: multi-scan (*SADABS*; Bruker, 2005)	*h* = −12→15
*T*_min_ = 0.962, *T*_max_ = 0.994	*k* = −20→24
13335 measured reflections	*l* = −12→12

### Refinement

**Table:**

Refinement on *F*^2^	Primary atom site location: structure-invariant direct methods
Least-squares matrix: full	Secondary atom site location: difference Fourier map
*R*[*F*^2^ > 2σ(*F*^2^)] = 0.063	Hydrogen site location: inferred from neighbouring sites
*wR*(*F*^2^) = 0.156	H atoms treated by a mixture of independent and constrained refinement
*S* = 1.03	*w* = 1/[σ^2^(*F*_o_^2^) + (0.0535*P*)^2^ + 0.8134*P*] where *P* = (*F*_o_^2^ + 2*F*_c_^2^)/3
4511 reflections	(Δ/σ)_max_ = 0.001
215 parameters	Δρ_max_ = 0.38 e Å^−^^3^
0 restraints	Δρ_min_ = −0.30 e Å^−^^3^

### Special details

**Table:**

Experimental. The crystal was placed in the cold stream of an Oxford Cryosystems Cobra open-flow nitrogen cryostat (Cosier & Glazer, 1986) operating at 120.0 (1) K.
Geometry. All e.s.d.'s (except the e.s.d. in the dihedral angle between two l.s. planes) are estimated using the full covariance matrix. The cell e.s.d.'s are taken into account individually in the estimation of e.s.d.'s in distances, angles and torsion angles; correlations between e.s.d.'s in cell parameters are only used when they are defined by crystal symmetry. An approximate (isotropic) treatment of cell e.s.d.'s is used for estimating e.s.d.'s involving l.s. planes.
Refinement. Refinement of *F*^2^ against ALL reflections. The weighted *R*-factor *wR* and goodness of fit *S* are based on *F*^2^, conventional *R*-factors *R* are based on *F*, with *F* set to zero for negative *F*^2^. The threshold expression of *F*^2^ > σ(*F*^2^) is used only for calculating *R*-factors(gt) *etc*. and is not relevant to the choice of reflections for refinement. *R*-factors based on *F*^2^ are statistically about twice as large as those based on *F*, and *R*-factors based on ALL data will be even larger.

### Fractional atomic coordinates and isotropic or equivalent isotropic displacement parameters (Å^2^)

**Table:**

	*x*	*y*	*z*	*U*_iso_*/*U*_eq_	
O1	0.37635 (19)	0.62888 (9)	0.70678 (18)	0.0317 (4)	
O2	0.04382 (17)	0.43778 (8)	0.70161 (18)	0.0258 (4)	
O3	0.06325 (15)	0.15459 (8)	0.69306 (16)	0.0186 (3)	
O4	0.25884 (15)	0.16149 (8)	0.56105 (17)	0.0190 (3)	
N1	0.2864 (2)	0.44806 (10)	0.3926 (2)	0.0188 (4)	
C1	0.3224 (2)	0.51866 (11)	0.3388 (2)	0.0169 (4)	
C2	0.3287 (2)	0.52803 (12)	0.1791 (3)	0.0200 (5)	
H2A	0.3127	0.4847	0.1091	0.024*	
C3	0.3577 (2)	0.59951 (13)	0.1230 (3)	0.0227 (5)	
H3A	0.3602	0.6050	0.0144	0.027*	
C4	0.3837 (2)	0.66416 (13)	0.2242 (3)	0.0261 (5)	
H4A	0.4023	0.7135	0.1847	0.031*	
C5	0.3817 (2)	0.65508 (13)	0.3821 (3)	0.0253 (5)	
H5A	0.4008	0.6984	0.4517	0.030*	
C6	0.3519 (2)	0.58298 (12)	0.4419 (2)	0.0193 (5)	
C7	0.3519 (2)	0.57481 (12)	0.6125 (3)	0.0210 (5)	
C8	0.3224 (2)	0.49397 (12)	0.6637 (2)	0.0206 (5)	
H8A	0.2854	0.4977	0.7585	0.025*	
H8B	0.4025	0.4634	0.6929	0.025*	
C9	0.2289 (2)	0.45255 (11)	0.5313 (2)	0.0185 (4)	
H9A	0.1501	0.4854	0.5017	0.022*	
C10	0.1895 (2)	0.37290 (11)	0.5803 (2)	0.0169 (4)	
C11	0.0934 (2)	0.36814 (11)	0.6637 (2)	0.0197 (5)	
C12	0.0477 (2)	0.29603 (11)	0.7021 (2)	0.0188 (5)	
H12A	−0.0207	0.2936	0.7552	0.023*	
C13	0.1030 (2)	0.22825 (11)	0.6622 (2)	0.0169 (4)	
C14	0.2058 (2)	0.23196 (11)	0.5876 (2)	0.0159 (4)	
C15	0.2472 (2)	0.30401 (11)	0.5455 (2)	0.0173 (4)	
H15A	0.3156	0.3065	0.4925	0.021*	
C16	−0.0213 (3)	0.43603 (13)	0.8271 (3)	0.0270 (5)	
H16A	−0.0456	0.4892	0.8499	0.041*	
H16B	0.0349	0.4139	0.9221	0.041*	
H16C	−0.0983	0.4038	0.7961	0.041*	
C17	−0.0519 (2)	0.14953 (12)	0.7498 (3)	0.0246 (5)	
H17A	−0.0727	0.0946	0.7626	0.037*	
H17B	−0.1220	0.1743	0.6732	0.037*	
H17C	−0.0399	0.1762	0.8523	0.037*	
C18	0.3688 (2)	0.16518 (12)	0.4928 (3)	0.0213 (5)	
H18A	0.4007	0.1123	0.4825	0.032*	
H18B	0.4357	0.1962	0.5615	0.032*	
H18C	0.3450	0.1895	0.3877	0.032*	
H1N1	0.241 (3)	0.4165 (16)	0.317 (3)	0.037 (8)*	

### Atomic displacement parameters (Å^2^)

**Table:**

	*U*^11^	*U*^22^	*U*^33^	*U*^12^	*U*^13^	*U*^23^
O1	0.0502 (13)	0.0223 (8)	0.0222 (8)	−0.0092 (8)	0.0077 (8)	−0.0045 (6)
O2	0.0385 (11)	0.0150 (7)	0.0295 (9)	0.0050 (7)	0.0196 (8)	0.0012 (6)
O3	0.0240 (9)	0.0132 (7)	0.0206 (7)	−0.0036 (6)	0.0093 (7)	0.0000 (6)
O4	0.0218 (9)	0.0126 (7)	0.0237 (8)	0.0007 (6)	0.0077 (7)	0.0000 (6)
N1	0.0262 (11)	0.0151 (8)	0.0161 (9)	−0.0046 (7)	0.0070 (8)	−0.0002 (7)
C1	0.0160 (11)	0.0151 (9)	0.0205 (10)	0.0004 (8)	0.0062 (9)	0.0006 (8)
C2	0.0206 (12)	0.0191 (10)	0.0222 (11)	−0.0015 (8)	0.0088 (9)	−0.0025 (8)
C3	0.0228 (13)	0.0263 (11)	0.0213 (11)	−0.0021 (9)	0.0094 (10)	0.0012 (9)
C4	0.0332 (15)	0.0187 (10)	0.0289 (12)	−0.0064 (10)	0.0124 (11)	0.0016 (9)
C5	0.0318 (15)	0.0188 (10)	0.0270 (12)	−0.0078 (9)	0.0101 (11)	−0.0022 (9)
C6	0.0213 (12)	0.0169 (10)	0.0201 (10)	−0.0030 (8)	0.0055 (9)	−0.0007 (8)
C7	0.0230 (13)	0.0193 (10)	0.0212 (11)	−0.0037 (9)	0.0059 (9)	−0.0008 (8)
C8	0.0276 (13)	0.0180 (10)	0.0158 (10)	−0.0007 (9)	0.0040 (9)	0.0006 (8)
C9	0.0234 (13)	0.0145 (9)	0.0187 (10)	0.0005 (8)	0.0070 (9)	0.0012 (7)
C10	0.0214 (12)	0.0134 (9)	0.0160 (9)	−0.0009 (8)	0.0043 (9)	0.0006 (7)
C11	0.0266 (13)	0.0141 (9)	0.0184 (10)	0.0009 (8)	0.0051 (9)	−0.0009 (8)
C12	0.0223 (13)	0.0181 (10)	0.0174 (10)	0.0010 (8)	0.0077 (9)	0.0017 (8)
C13	0.0235 (12)	0.0134 (9)	0.0129 (9)	−0.0023 (8)	0.0018 (8)	0.0014 (7)
C14	0.0212 (12)	0.0129 (9)	0.0128 (9)	0.0012 (8)	0.0019 (8)	−0.0013 (7)
C15	0.0194 (12)	0.0164 (9)	0.0165 (10)	−0.0004 (8)	0.0044 (9)	0.0016 (8)
C16	0.0357 (15)	0.0231 (11)	0.0262 (12)	0.0103 (10)	0.0154 (11)	0.0034 (9)
C17	0.0275 (14)	0.0198 (11)	0.0294 (12)	−0.0029 (9)	0.0125 (10)	0.0019 (9)
C18	0.0224 (13)	0.0192 (10)	0.0250 (11)	0.0013 (9)	0.0111 (10)	0.0000 (8)

### Geometric parameters (Å, º)

**Table:**

O1—C7	1.224 (2)	C8—C9	1.519 (3)
O2—C11	1.377 (2)	C8—H8A	0.9900
O2—C16	1.417 (3)	C8—H8B	0.9900
O3—C13	1.379 (2)	C9—C10	1.518 (3)
O3—C17	1.432 (3)	C9—H9A	1.0000
O4—C14	1.377 (2)	C10—C11	1.387 (3)
O4—C18	1.435 (3)	C10—C15	1.398 (3)
N1—C1	1.383 (3)	C11—C12	1.398 (3)
N1—C9	1.470 (3)	C12—C13	1.384 (3)
N1—H1N1	0.90 (3)	C12—H12A	0.9500
C1—C2	1.407 (3)	C13—C14	1.400 (3)
C1—C6	1.409 (3)	C14—C15	1.389 (3)
C2—C3	1.380 (3)	C15—H15A	0.9500
C2—H2A	0.9500	C16—H16A	0.9800
C3—C4	1.401 (3)	C16—H16B	0.9800
C3—H3A	0.9500	C16—H16C	0.9800
C4—C5	1.379 (3)	C17—H17A	0.9800
C4—H4A	0.9500	C17—H17B	0.9800
C5—C6	1.405 (3)	C17—H17C	0.9800
C5—H5A	0.9500	C18—H18A	0.9800
C6—C7	1.482 (3)	C18—H18B	0.9800
C7—C8	1.511 (3)	C18—H18C	0.9800
			
C11—O2—C16	116.60 (16)	C8—C9—H9A	107.9
C13—O3—C17	116.79 (16)	C11—C10—C15	118.63 (18)
C14—O4—C18	116.03 (15)	C11—C10—C9	118.94 (18)
C1—N1—C9	115.41 (16)	C15—C10—C9	122.42 (19)
C1—N1—H1N1	115.3 (16)	O2—C11—C10	116.43 (18)
C9—N1—H1N1	111.6 (17)	O2—C11—C12	122.38 (19)
N1—C1—C2	120.58 (18)	C10—C11—C12	121.16 (19)
N1—C1—C6	120.86 (18)	C13—C12—C11	119.4 (2)
C2—C1—C6	118.56 (18)	C13—C12—H12A	120.3
C3—C2—C1	120.75 (19)	C11—C12—H12A	120.3
C3—C2—H2A	119.6	O3—C13—C12	123.56 (19)
C1—C2—H2A	119.6	O3—C13—C14	116.19 (18)
C2—C3—C4	120.83 (19)	C12—C13—C14	120.25 (18)
C2—C3—H3A	119.6	O4—C14—C15	124.66 (19)
C4—C3—H3A	119.6	O4—C14—C13	115.80 (17)
C5—C4—C3	118.9 (2)	C15—C14—C13	119.53 (18)
C5—C4—H4A	120.5	C14—C15—C10	120.82 (19)
C3—C4—H4A	120.5	C14—C15—H15A	119.6
C4—C5—C6	121.3 (2)	C10—C15—H15A	119.6
C4—C5—H5A	119.3	O2—C16—H16A	109.5
C6—C5—H5A	119.3	O2—C16—H16B	109.5
C5—C6—C1	119.58 (19)	H16A—C16—H16B	109.5
C5—C6—C7	120.06 (19)	O2—C16—H16C	109.5
C1—C6—C7	120.37 (18)	H16A—C16—H16C	109.5
O1—C7—C6	122.93 (19)	H16B—C16—H16C	109.5
O1—C7—C8	121.87 (18)	O3—C17—H17A	109.5
C6—C7—C8	115.19 (17)	O3—C17—H17B	109.5
C7—C8—C9	110.81 (17)	H17A—C17—H17B	109.5
C7—C8—H8A	109.5	O3—C17—H17C	109.5
C9—C8—H8A	109.5	H17A—C17—H17C	109.5
C7—C8—H8B	109.5	H17B—C17—H17C	109.5
C9—C8—H8B	109.5	O4—C18—H18A	109.5
H8A—C8—H8B	108.1	O4—C18—H18B	109.5
N1—C9—C10	112.03 (16)	H18A—C18—H18B	109.5
N1—C9—C8	108.16 (18)	O4—C18—H18C	109.5
C10—C9—C8	112.88 (17)	H18A—C18—H18C	109.5
N1—C9—H9A	107.9	H18B—C18—H18C	109.5
C10—C9—H9A	107.9		
			
C9—N1—C1—C2	−153.9 (2)	N1—C9—C10—C15	25.1 (3)
C9—N1—C1—C6	25.2 (3)	C8—C9—C10—C15	−97.3 (2)
N1—C1—C2—C3	176.4 (2)	C16—O2—C11—C10	−160.0 (2)
C6—C1—C2—C3	−2.7 (3)	C16—O2—C11—C12	21.9 (3)
C1—C2—C3—C4	1.0 (4)	C15—C10—C11—O2	177.03 (19)
C2—C3—C4—C5	1.0 (4)	C9—C10—C11—O2	−2.5 (3)
C3—C4—C5—C6	−1.2 (4)	C15—C10—C11—C12	−4.8 (3)
C4—C5—C6—C1	−0.5 (4)	C9—C10—C11—C12	175.6 (2)
C4—C5—C6—C7	179.3 (2)	O2—C11—C12—C13	−179.2 (2)
N1—C1—C6—C5	−176.7 (2)	C10—C11—C12—C13	2.8 (3)
C2—C1—C6—C5	2.5 (3)	C17—O3—C13—C12	8.6 (3)
N1—C1—C6—C7	3.5 (3)	C17—O3—C13—C14	−171.93 (18)
C2—C1—C6—C7	−177.4 (2)	C11—C12—C13—O3	−179.02 (19)
C5—C6—C7—O1	0.5 (4)	C11—C12—C13—C14	1.6 (3)
C1—C6—C7—O1	−179.7 (2)	C18—O4—C14—C15	3.5 (3)
C5—C6—C7—C8	−178.2 (2)	C18—O4—C14—C13	−176.60 (18)
C1—C6—C7—C8	1.6 (3)	O3—C13—C14—O4	−3.1 (3)
O1—C7—C8—C9	148.3 (2)	C12—C13—C14—O4	176.39 (19)
C6—C7—C8—C9	−33.0 (3)	O3—C13—C14—C15	176.84 (18)
C1—N1—C9—C10	178.76 (19)	C12—C13—C14—C15	−3.7 (3)
C1—N1—C9—C8	−56.2 (2)	O4—C14—C15—C10	−178.52 (19)
C7—C8—C9—N1	59.1 (2)	C13—C14—C15—C10	1.6 (3)
C7—C8—C9—C10	−176.39 (18)	C11—C10—C15—C14	2.6 (3)
N1—C9—C10—C11	−155.4 (2)	C9—C10—C15—C14	−177.8 (2)
C8—C9—C10—C11	82.2 (3)		

### Hydrogen-bond geometry (Å, º)

*Cg*1 and *Cg*2 are the centroids of the C1–C6 and C10–C15 rings, respectively.

**Table:**

*D*—H···*A*	*D*—H	H···*A*	*D*···*A*	*D*—H···*A*
N1—H1*N*1···O3^i^	0.90 (3)	2.32 (3)	3.156 (2)	155 (3)
C2—H2*A*···O4^i^	0.95	2.59	3.439 (3)	150
C16—H16*B*···O3^ii^	0.98	2.58	3.459 (3)	150
C8—H8*B*···*Cg*1^iii^	0.99	2.74	3.698 (2)	164
C16—H16*C*···*Cg*1^iv^	0.98	2.68	3.518 (3)	144
C17—H17*C*···*Cg*2^ii^	0.98	2.76	3.560 (3)	140
C18—H18*C*···*Cg*2^i^	0.98	2.75	3.574 (3)	142

Symmetry codes: (i) *x*, −*y*+1/2, *z*−1/2; (ii) *x*, −*y*+1/2, *z*+1/2; (iii) −*x*+1, −*y*+1, −*z*+1; (iv) −*x*, −*y*+1, −*z*+1.
